# Lifting lockdown: Renewed access to arts and cultural activities

**DOI:** 10.1186/s12889-022-14282-7

**Published:** 2022-10-10

**Authors:** Joanne Worsley, Josie Billington, Ekaterina Balabanova, Melissa Chapple

**Affiliations:** 1 Department of Primary Care and Mental Health, University of Liverpool, Liverpool, UK; 2 Department of English, University of Liverpool, Liverpool, UK; 3 Department of Communication and Media, University of Liverpool, Liverpool, UK

**Keywords:** COVID-19, Renewed access, Re-connecting, Hybridity, Public mental health, Wellbeing, Social prescribing

## Abstract

**Background:**

The effects of COVID-19 on mental health are profound. While there is a growing body of evidence on arts supporting mental health, the re-engagement with in-person arts and cultural activity has remained slow following the lifting of restrictions.

**Methods:**

Interviews with 14 representatives, including providers and practitioners, from 12 arts and cultural organisations within the Liverpool City Region (LCR) were conducted. The aim was to examine the impact of COVID-19 restrictions easing on arts and cultural provision in the LCR, and on the mental health and wellbeing of those whom arts and cultural organisations serve, including those who would usually access arts through formal healthcare routes (e.g., those usually served via arts organisations’ partnership with health or social care providers). Data were analysed using framework analysis.

**Results:**

Three overarching themes were identified: The new normal: ‘Out of crisis comes innovation’; Complexities of operating ‘in the new COVID world’; and Reimagining arts in mental healthcare.

**Conclusion:**

As engagement in community and cultural activities plays a public health role, a hybrid delivery of arts and culture – ensuring continued online access alongside in-person provision – will be vital for people’s recovery. Alongside efforts to reimagine arts in mental healthcare in the wake of the crisis caused by the pandemic, the role of arts and culture in providing stigma-free environments to reconnect the vulnerable and isolated is more critical than ever. Recommendations on the role of arts and culture in sustaining the mental health and wellbeing of the population and embedding the arts within clinical care and public health prevention schemes are provided.

**Supplementary Information:**

The online version contains supplementary material available at 10.1186/s12889-022-14282-7.

## Introduction

The effects of COVID-19 on mental health and wellbeing are likely to be profound [1, 2]. The Centre for Mental Health has estimated that there will be an extra 10 million people in need of new or additional mental health support in England as a direct consequence of the pandemic [3]. Alongside limited mental health resources available, there are projected long-lasting effects of the COVID-19 crisis, especially for vulnerable groups, such as those with pre-existing mental health difficulties [1].

There is a growing body of literature suggesting that the arts can support mental health [4]. Research has demonstrated protective associations between arts participation (e.g., actively engaging in arts activities such as singing or dancing) or cultural engagement (e.g., accessing museums or theatres) and both the prevention and management of mental ill health [5–7]. As such, the arts have been conceptualised as multimodal health activities, combining multiple different factors known to be beneficial to health, such as physical activity, social interaction, and cognitive stimulation. Contributing further to their salutogenic effects, the arts involve evocation of the imagination and aesthetic engagement, thereby providing an inherent motivation for participation [8]. Beyond individual benefits, such as emotional expression and skill acquisition, arts engagement in groups facilitates social capital and social cohesion within communities [4]. Salutogenic approaches are therefore important in the treatment and prevention of long-term conditions, and have the potential to reduce pressure on healthcare systems [9].

During the COVID-19 pandemic, individuals with pre-existing mental health conditions experienced poor mental health and were unable to engage in activities that usually protected their mental health, such as visiting museums and theatres [10]. Rapid innovation in the arts and cultural sector accelerated digitalisation, and online arts provision became a lifeline for those with pre-existing mental health difficulties by restoring wellbeing and reducing feelings of isolation [11]. Although in-person arts and cultural provision became accessible following the conclusion of lockdown, re-engagement remains slow due to a continued sense of risk to health [12]. There has been limited research exploring engagement with these venues following the lifting of restrictions [13].

With some of the poorest mental health outcomes in the country [14], and one of the richest concentrations of culture in the UK [15], the Liverpool City Region (LCR) has a pioneering history of harnessing arts for mental health care through partnerships between arts and health care providers [16–18]. Arts-in-health partnerships are unique and wide-ranging in the LCR. One National Health Service (NHS) Foundation Trust, a provider of adult mental health services across North West England, has nurtured a number of creative and cultural partnerships, which are now integral elements of the care offer. A civic concert hall, for example, has been working in partnership with this particular NHS Trust for over 14 years on a music in mental health programme, delivering courses across a range of clinical and community settings [17]. Another NHS Trust has commissioned the practice of shared reading, delivered by a Liverpool-based outreach charity, for chronic pain patients since 2014. The pain clinic at this Trust now offers a pain-management programme for patients which extends beyond cognitive behavioural therapy. These examples show two sectors imaginatively pooling resources to address the health needs of a disadvantaged population.

This study aimed to examine the impact of COVID-19 restrictions easing on arts and cultural provision in the LCR, and on the mental health and wellbeing of those whom arts and cultural organisations serve, including those who would usually access arts through formal healthcare routes (e.g., those usually served via arts organisations’ partnership with health or social care providers). Through delivering community programmes, arts providers and practitioners are well-placed to reflect on the impact of such provision on beneficiaries’ mental health and wellbeing.

## Methods

This study is part of a larger longitudinal project examining the impact on arts providers, practitioners, and beneficiaries of restricted access to arts and cultural activity, and the impact of renewed access to ‘new normal’ arts and cultural provision. While we did not collect any data pertaining to organisation or practitioner support, the Department for Culture, Media, and Sport (DCMS) announced a £1.57 billion Culture Recovery Fund (CRF)^1^ in response to the COVID-19 pandemic. The CRF fund aimed to support arts and cultural organisations by offering financial security and the opportunity to rethink and strategize [19]. Qualitative semi-structured interviews were conducted at two waves: wave 1 took place during a period of national and local restrictions (September 2020 to May 2021; see 11) and wave 2 took place after all restrictions were lifted in July 2021 (August to October 2021). Wave 1 interviews were conducted between September 2020 and May 2021 covering a period during which arts and cultural provision in LCR remained largely online. The findings from wave 1 have been reported elsewhere (see 11). This paper focuses on the accounts of arts providers and practitioners after the lifting of restrictions in July 2021, as beneficiaries returned to in-person provision (reporting findings from wave 2 data collection only).

### Ethical approval

Ethical approval was received from the Central University Research Ethics Committee (7994; see 11). All methods were carried out in accordance with relevant guidelines and regulations (see 11). Informed consent was obtained from all participants.

### Participants

Fourteen representatives (13 females and 1 male) including providers and practitioners from 12 arts and cultural organisations within the LCR - museums (*n* = 1), theatres (*n* = 2), galleries (*n* = 5), concert halls (*n* = 2), community and participatory arts organisations (*n* = 4) - participated in this wave. These organisations run participatory programmes and community-focused arts activities outside of conventional arts provision (public concerts, plays, exhibitions), targeting community (often vulnerable) participants, often in collaboration with health and/or social care providers. For the whole longitudinal study, civic arts organisations in the Liverpool City Region (e.g., Liverpool Philharmonic, National Museums Liverpool, Tate Liverpool) were approached to participate, with additional arts organisations being identified and recruited through snowballing.

### Data collection

Interviews were carried out between August and October 2021, after the lifting of restrictions in England on 19th July 2021. Arts organisations had different priorities and safety measures in place. Interviews followed a semi-structured set of questions. The topic guide comprised questions exploring current provision, with a focus on renewed access to in-person provision as well as continued online arts and cultural provision; the impact of renewed accessibility on beneficiaries’ mental health and wellbeing; and the role of arts and culture during the future recovery period (see Supplementary file 1). Interview duration ranged from 30 min to one hour. All interviews were recorded and transcribed verbatim.

### Analysis

Data were analysed using framework analysis. As framework analysis reduces data loss, it was selected as appropriate [20]. Framework analysis also allows for a combined deductive and inductive approach to data analysis [21]. The procedure of framework analysis advocated by Gale et al. [21] was followed. Familiarisation involved the process of transcription, followed by repeated viewing of the transcripts and audio recordings. Line-by-line coding, derived from a largely inductive approach, ensured that data were not overlooked. A subset of transcripts were coded independently by two researchers. Codes were categorised and defined to produce an analytical framework. The framework was applied to all transcripts through indexing. The resultant themes and subthemes were checked by the team. Themes were renamed and refined until consensus was reached.

## Results

Three overarching themes were identified: The new normal: ‘Out of crisis comes innovation’; Complexities of operating ‘in the new COVID world’; and Reimagining arts in mental healthcare. A number of subthemes were identified in relation to each overarching theme (see Table 1).

**Table 1:** Overarching themes and subthemes

Themes	*Subthemes*
The new normal: ‘Out of crisis comes innovation’	*Returning to in-person provision*
	*Continuing online provision*
Complexities of operating ‘in the new COVID world’	*New concerns*
	*Future uncertainty*
Reimagining arts in mental healthcare	*Culture and arts in social prescribing*
	*Arts in health settings*

### Theme 1: The new normal: ‘Out of crisis comes innovation’

#### Returning to in-person provision

During the COVID-19 pandemic, in-person arts and cultural engagement, upon which a significant number of vulnerable populations relied for regular contact, were ceased. As people were deprived of opportunities previously taken for granted, arts providers and practitioners described the first in-person gathering following the conclusion of lockdown as ‘*absolutely joyous*’ (Participant 1, Dance organisation), a ‘*celebration*’ (Participant 9, Musician), with beneficiaries experiencing positive emotions upon re-engaging with others in-person:

*When that live recording weekend happened, which was the last weekend of August… It was just like a big party… the joy, the over joy of the young people getting back with each other was just something really, really special* (Participant 9, Musician).

Some, however, felt apprehensive, particularly the elderly and those with underlying health conditions. In-person engagement involves risk assessment, for both arts providers and beneficiaries. Amongst customary beneficiaries, there remained a sense of caution, anxiety, and risk around re-engaging in-person, particularly in indoor spaces:

*As we’ve gone to larger groups and restrictions have eased off a little bit where we can do that, there is definitely still an anxiety to doing that and a mixture of energies in the room from those who are anxious about returning and those who are incredibly enthusiastic. That juxtaposition of attitudes can create sometimes not the most smoothest running session I think in these new COVID times* (Participant 10, Music organisation).

In this context and as many arts organisations cater for vulnerable populations, COVID-19 safety measures, such as social distancing and face coverings, remained in-place:

*Because we’re working with a lot of vulnerable adults and young people, we’ve been using the same [face-to-face] COVID procedures that we had pre things becoming more open in July… masks, social distancing, we don’t provide refreshments* (Participant 10, Music organisation).

*These young people have very individualised care plans and it’s very rare in the week that they do a group activity [in an NHS in-patient setting] because that is very challenging for them… So pre-COVID, it was a real task and always a bit of a challenge every week to gather the young people around the table, and then post-COVID when we resumed, we had to observe social distancing measures… I’d have an average of five young people… each on one desk. So that made that task of bringing people together even more difficult* (Participant 9, Musician).

The importance of trusting organisations to ensure COVID safety during in-person provision was acknowledged. Continued clear communication and messaging about safety measures implemented by organisations was necessary to mitigate some of the anxieties that vulnerable people feel in relation to returning to in-person provision:

*There is a continued nervousness about that [referring to older people returning to in-person provision]. So, we have to make sure that we give that reassurance that it’s safe to connect with House of Memories… I think we will continue to do that* (Participant 2, Museum).

*They’ve [referring to a chronic pain reading group] decided that they’re going to meet up and have an afternoon tea at Calderstones. I think it’s partly because it’s seen as a safe environment… I imagine that when you are constantly managing your health, you maybe feel like you need to have trust in the organisations that are around you, particularly with a risk that needs to be so carefully controlled* (Participant 8, Shared reading organisation).

As some beneficiaries were reluctant to share their engagement preference, or preferences could be ‘hard to gauge’ (‘*you’re never sure if people are just being polite and saying they are fine when they’re not’* (Participant 14, Photography gallery)), one solution implemented by a small arts organisation was to circulate a ‘COVID survey’ anonymously seeking participants’ views and based on that develop their own risk assessment. This allowed them to combine online and in-person engagement:

*We’ve got a COVID survey that we send out to the participants. On the basis of that survey, we develop our risk assessment… We basically bring people together when there’s either a big need within the participants or… when we think there’s real value to it in terms of the development of the project… I think in terms of wellbeing and mental health, if people are happy to, they get a lot out of being in the room. So, for example there’s a project that we run called Above and Beyond, which works in Knowsley, Bootle and Birkenhead. Those groups have weekly sessions in the online space, but then once a month, we will bring them together to do specific either issue exploration or skills development activities*… *We’re really mindful of the fact there are many people out there that aren’t comfortable coming back into the space for different reasons. So, we’re trying to make sure that nobody’s left behind in terms of how we deliver our provision moving forward* (Participant 7, Theatre).

Given the juxtaposition of attitudes, some arts organisations offered engagement opportunities in alternative spaces. For example, higher transmissibility in indoor spaces resulted in arts organisations bringing participants together in outdoor areas. Some organisations viewed this as an opportunity to involve new audiences, as engagement in streets or parks may feel more accessible to those who do not feel comfortable accessing arts venues or unfamiliar arts forms:

*We’re actually [doing] the men’s group and the women’s group outdoors… We just thought it’d be a nice way to celebrate dance and celebrate being together, but just do it in an open space and see if anyone else wants to join in* (Participant 1, Dance organisation).

Arts providers and practitioners have also started offering services within familiar community spaces to engage a wider range of people. Organisations have therefore reimagined themselves in order to better serve society’s needs, delivering provision in alternative venues, such as libraries and supermarket carparks:

*We are working with… the council and the libraries network, taking workshops to the libraries to reach communities that might have barriers. Sometimes just the thought of coming into a dance studio is a barrier, or going into town… We are going to start small with four or five libraries… see how it goes, and then we can expand it to reach more people* (Participant 1, Dance organisation).

*The other new initiative that we have just gone live with is called House of Memories On the Road… On the Road is a 30 square foot, immersive cinema and activity space. It can drive into local community settings, into neighbourhoods, it can work with voluntary sector groups, with primary care networks, it can go to a hospital trust, it could be in a GP carpark or a supermarket, and the idea is that we bring the museum to you, where you are*…*With Local Authorities support, we can identify those neighbourhoods that have least opportunity, and perhaps those groups of elders who are the most socially isolated or older people that have experienced loneliness. We can target specific groups and that makes it really responsive as an experience* (Participant 2, Museum).

New collaborations, such as those between museums and local authorities, have therefore created new ways of working to support vulnerable, marginalised, or isolated members of communities. Taken together, the focus was not on returning to ‘normal’ provision, but instead on finding ways to adapt or reimagine conventional provision. Arts providers and practitioners referred to this transitionary phase as a period of readjustment, with participants and staff adapting and ‘*adjusting to this as being the new normal*’ (Participant 10, Music organisation):

*For many people who have had really, really tough experiences, real challenges because of COVID, to readapt to this is like another huge change. We had a huge change two years ago and now we have to go through another huge change. I think this readaptation could nearly be as important for some people as the adaptation when we had to go into lockdown* (Participant 9, Musician).

The importance of the arts during this transition period was highlighted in relation to helping people ‘*to build a sense of routine and a sense of purpose*’ (Participant 4, Concert Hall), which is always an important consideration for people experiencing mental health difficulties. Although there is a continued nervousness amongst vulnerable populations, in-person arts provision provides ‘*meaningful activity*’, and participation may inspire and enable people to feel confident about returning to other in-person events and activities in the city centre. In this respect, in-person arts engagement has been perceived as a ‘*steppingstone*’:

*I think that city centre cultural scene could play a massive part in bringing people who are less keen to come back, people who are still finding it really challenging to get back involved in things because they’re still very, very worried about COVID. The shops and going for a meal aren’t going to be enough to tempt them out but something that’s more meaningful to them, like coming to an exhibition or a workshop, where they can get involved in something that they love doing or going to the theatre or a concert. All of those experiences are going to be really important to getting people back out and engaged again, particularly when you think about more vulnerable groups, many of whom will be particularly cautious. Some of our Blue Room members probably still wouldn’t really be going out if it wasn’t for Blue Room. Because they’re motivated enough by that and they really want to come back, that’s a steppingstone hopefully to other things for them* (Participant 5, Contemporary arts centre).

The importance of in-person arts and cultural engagement as a catalyst for providing a sense of community through shared collective experiences was highlighted by arts providers and practitioners. They talked of an ‘*overall sense of comfort, of togetherness, of coming together’* especially as ‘*we’ve all been through periods of isolation*’ (Participant 9, Musician):

*Being physically in a space together, being at [name of organisation] is being part of a bigger community of people… To see Blue Room members coming back in, and just being able to be around this [platform installation] and to be in a place where there’s something beautiful to look at when there’s other people around and children and families around and be back as part of our community again. It looked like people are really enjoying it… During lockdown… a lot of our Blue Room members would have been very much at home, not being anywhere else at all… It’s probably going to have a huge impact on people being back in a community, because Blue Room members have always been a real part of [name of organisation] community* (Participant 5, Contemporary arts centre).

*With the dance for Parkinson’s, they have been engaged online with us for a while but there would be times during the proper lockdown where dance for Parkinson’s participants were telling us that their conditions have worsened, because they couldn’t come into the studio and get that connection and that movement. So, I think that will make a huge, huge difference. I think it was the connection that they were missing… It’s easy to follow instructions on the screen, but it’s just not the same because especially in the dance for Parkinson’s classes, we have live music, volunteers, people can bring their carers or their family and it’s such a joyous atmosphere* (Participant 1, Dance organisation).

During the COVID-19 pandemic, usual ways of interacting and obtaining social support were also disrupted. In-person provision provides the opportunity to interact, connect with others, and obtain social support, thereby boosting community connectedness and a sense of collective healing:

*Nothing beats that in-person connection*… *One of the groups lost someone during the pandemic and had they have been coming to in-person classes, they would have been able to deal with it together, and I think it’s quite healing for them to all be together again* (Participant 1, Dance organisation).

In line with this, there is a revitalised appreciation of the humanising and connective power of arts and culture, particularly a new sense of their value for processing the trauma and the negative emotions spawned by the COVID-19 pandemic:

*It’s an opportunity to reflect on the last 18 months as well because when you’re in it, you can’t really see what it is. But when you look back on it, it has been such a massive thing in people’s lives. It’s not you open the door and go outside and suddenly it’s all better; you’ve been through quite a traumatic experience. So, there’s an element of recovery that’s going on at the moment, which is slow* (Participant 7, Theatre).

Ancillary social and relational qualities enrich in-person provision. For example, sharing spontaneous one-on-one conversations before or following social bonding activities, such as singing or dancing - ‘*the little conversations that you have over a cup of tea, the one-to-one check ins’* (Participant 7, Theatre), ‘*a little chat beforehand… a chat afterwards*’ (Participant 1, Dance organisation) - enhance collective experiences. Interacting with others online often does not provide the same opportunities for physical intimacy, when compared to being together in a shared physical space:

*When you’re in a room with people, there’s a connection that happens, bodies align, we align with each other, we learn about each other, we take emotional and physical cues from each other without even recognising that and there is a way of syncing our bodies not by physically touching, just by being in a room together* (Participant 3, Theatre).

*Connecting with people I think is the one that’s probably always the most important to our Blue Room members as well as being physically active because a lot of people can end up very sedentary at home and physically that can take a toll on people… So just getting back out and walking around rather than just sitting all day is going to make a big difference to people’s health directly, as well as the impact on their wellbeing* (Participant 5, Contemporary arts centre).

#### Continuing online provision

The embedding of a hybrid delivery of arts and culture – ensuring continued online access alongside in-person provision – appears to meet public demand as many beneficiaries remain hesitant about re-engaging in-person. Arts providers were keen to ensure that their ‘*programme is fully accessible*’ (Participant 11, Cultural and creative hub) by providing a range of engagement opportunities, necessitated by the need to ensure that ‘*no one is left behind*’ (Participant 7, Theatre):

*We have learned that a blend of both in-person and online activity is beneficial to provide a range of points of access for people to participate on their terms, so that they have choice* (Participant 4, Concert Hall).

*We’ve learned that we probably need to keep all options open, like all different avenues for ways of engaging with people… We’re going to be having a blended delivery, tentatively moving more to physical with less digital, but digital always still being there* (Participant 14, Photography gallery).

As online provision remained vital for many, arts and cultural organisations explored creative means of integrating online and in-person provisions, which afforded beneficiaries (as well as practitioners) the option of attending a session either in-person or remotely:

*We always set up… a hybrid of Zoom and face to face work… a Zoom link, and a big screen with a mic and an audio interface so people can engage with it on Zoom if they choose not to attend… Last night’s session really benefitted from that, because the composer, who is working with young musicians on new music actually had COVID. So, he couldn’t attend the session and led it over Zoom… and also anyone else who didn’t attend could also access that link* (Participant 10, Music organisation).

*The hybrid option is probably our biggest success story, because it gives people the option to experience that in-person experience as it can feel like they are in the in-person class and see everyone and feel like they’re in the studio, but if for whatever reason they need to stay at home, then they can. It allows the in-person people to still connect with the people who can’t come as well* (Participant 1, Dance organisation).

Other arts providers perceived online provision as an enabler to people engaging in in-person provision. One described online provision as a ‘*springboard*’ or ‘*steppingstone*’ (Participant 3, Theatre), allowing people to engage in arts activities online before transferring to in-person provision. This made it possible for beneficiaries to familiarise themselves with the course and facilitator before engaging in-person, and may be an important step for those with mental health difficulties:

*In terms of the Life Rooms [community NHS service], we plan on continuing to have an online provision as long as they have the online platform… It’s a really accessible steppingstone to coming into the room*… *Doing drama online is great and there is definitely room for development and this beautiful space to be creative and to express ourselves and to try new things… It isn’t the same as being in a room. It can’t be, you’re stuck in a square or a rectangle. It isn’t a replacement. I suppose it’s a brilliant thing to have as a steppingstone to act as a springboard to support people to feel comfortable and confident coming to sessions and understanding what sessions look like* (Participant 3, Theatre).

The re-use of the notion of ‘steppingstone’ here further highlights the variety of ways in which arts and culture are important, especially for those with mental health difficulties. It is crucial to retain online provision for certain groups, such as those with physical or mental health conditions, who may find re-engaging with arts and culture in-person challenging:

*Even when they’re able to go back in person, which they’re taking very, very slowly because there’s lots of complex physical health difficulties in the group, COVID is such a risk. She [referring to a service user] said they want to keep an online group going and the in-person group because they have found that some people who could only patchily attend the in-person group pre COVID have had an almost 100% attendance rate during COVID… To have a service and activity that comes into your home, rather than you have to get out of bed and get to it, could be enormously beneficial*… *They’re saying, even when we can go back in person, we want to keep both these things [online and in person] because we want options. We want to be able to go and be with people when we can manage it, but we want to be able to access all these benefits when our physical health fails. It’s quite revolutionary to me when you think, wow, if we could have the time and the freedom to work in that way, what would people tell us that they needed? Everything breaking through COVID has actually allowed something else to happen, which has been something much nearer to innovation* (Participant 8, Shared reading organisation).

*It might cost to some people having to go out, having to pay for travel, having to face social challenges of getting on the bus… Social in the sense that if you’re in a group your social skills are very exposed… This feeling of suddenly having to face real life and be back in action and people expecting you to be like you were before COVID when we’ve all had loads of changes in our lives. I think for some of the participants, I’m referring mainly to the Life Rooms participants [users of a community NHS service], I can imagine how it could be challenging to come back and be expected to participate like the way they used to do* (Participant 9, Musician).

The flexibility of online provision meant that arts providers brought people together across the city region and country, which enabled beneficiaries to connect with likeminded others and feel part of a wider community than would otherwise be possible:

*Everyone’s realised the importance of putting mental health first. We’ve had a couple of things… where it was like… that doesn’t make sense for us, because it’s expensive, and we’re a really small team. Then when we were talking about it, we were like, actually, it will give this vulnerable group additional access to a community and a resource, and we put that before the money* (Participant 1, Dance organisation).

*Something else that struck me in what [name of beneficiary] said was the potential to meet people from other areas. She said that she’s really enjoyed going to other online shared reading groups that we’ve been running through the pandemic, as well as running her own. Because she said, I meet all these people from all over the UK, which, for any number of reasons, just wouldn’t be possible otherwise. So, what we mean by that notion of community becomes really interesting* (Participant 8, Shared reading organisation).

*If we’d had a session in Walton [Life Rooms, community NHS service], a session in Bootle [Life Rooms] and a session in Southport [Life Rooms], we would have three distinct groups of people who wouldn’t necessarily meet until we held a celebration event at the end of the project. Whereas now people from those areas and from other areas across the LCR can take part in one activity together* (Participant 4, Concert Hall).

Given the benefits of digital provision, such as inclusivity and accessibility, arts and cultural organisations intend to provide both in-person and digital provision moving forward, showing that digital provision is now an intrinsic part of strategic decision-making:

*When we began planning the new partnership with Clatterbridge Cancer Centre, for example, we planned online provision as a core part of the partnership alongside additional in-person activity because of the benefits around flexibility of time and accessibility for participants* (Participant 4, Concert Hall).

Nevertheless, such strategic decisions are likely to be dependent on the individual financial circumstances of both civic institutions and grass roots:

*What we’ve been able to do during COVID is to sufficiently adapt our programmes so we can support people digitally, face to face, we can come out to you, you can come into our venues. We’ve offered a wider range of engagement opportunity that, funding permitting and everything else, we are really determined to try and maintain* (Participant 2, Museum).

### Theme 2: Complexities of operating ‘in the new COVID world’

#### New concerns

Lack of funds and staff redundancies have rendered some arts organisations (both civic and grass roots) unable to operate at full capacity following the easing of restrictions:

*We would originally have been open seven days a week, but we’re still only able to afford to open for five* (Participant 5, Contemporary arts centre).

*Because we lost some staff, we don’t have enough people to meet demand. We’d love to be open as often and for as long as we could be during the week, but we’re still scaled back because we just don’t have the bodies to be there* (Participant 1, Dance organisation).

Returning to in-person provision has also created many practical and logistical issues. Co-ordinating in-person projects in the new COVID world has presented new challenges, resulting in new, additional concerns for arts providers:

*A lot of the schools have slightly different approaches to the COVID rules and regulations. Every person that we deal with has a slightly different attitude or differing feelings about the restrictions and the pandemic. We have our own risk assessments and our own safety procedures… Because there’s not one line of joined up thinking… keeping on top of all of those things as someone coordinating projects that are now coming back to face-to-face in this new world is to be quite frank a very, very anxious time, an incredibly challenging set of circumstances* (Participant 10, Music organisation).

These complexities are amplified further as many practitioners have either tested positive for COVID-19 or received notification of the need to self-isolate (colloquially known as being ‘pinged’)^2^, which has led to staff shortages, venue closures, and/or cancellations:

*Every facilitator that we’ve worked with, bar two of them… has either had COVID or been pinged at one point to be self-isolating… We have had sessions where I turned up to coordinate and run and set up … and had both of the facilitators unable to attend… because of being pinged. We then had a six-hour session happening in an hour with no facilitators to run the session. Obviously the same could go for PAs [Personal Assistants] who have been pinged, young people that have been pinged, young people could get COVID by being in close contact, that’s happened left, right and centre, and it’s totally unpredictable and it’s totally unprecedented* (Participant 10, Music organisation).

*It’s very difficult at the moment if one person tests positive, there’s a whole knock-on effect of that in terms of staff having isolate even though the government rules have changed around people isolating if they’ve been double vaccinated, the regulations within the adult social care sector are different. So, if you’re working with anybody who’s clinically extremely vulnerable, then that member of staff can’t come back to work with them, and we have clinically extremely vulnerable people in all of our Blue Room groups* (Participant 5, Contemporary arts centre).

*We had a show on at the Playhouse called Love Liverpool, which was a socially distanced rehearsal and performance, and yet still two of the members of the cast got COVID so we had to cancel the show. I think probably every show is at risk of that* (Participant 3, Theatre).

Many beneficiaries have also been notified by the NHS COVID-19 app to self-isolate. This was particularly obstructive in one youth theatre group who were in the middle of rehearsing as many young people were ‘*being pinged*’, resulting in ‘*recasting, recasting and recasting based on people having been told to self-isolate*’ (Participant 3, Theatre):

*In terms of our youth theatre provision, there’s been huge challenges making these shows up because, especially at the time when they were rehearsing to put the show on, there was a really high number of COVID cases in schools, and children were being expected to test on a regular basis. So, then there were more much higher numbers of positive results… So, it was a big challenge for our YEP [Young Everyman Playhouse] team in terms of putting that show on just because they were really up against it with people having to be isolated or being pinged*. *One of the directors had to isolate for two weeks, because his little boy got COVID* (Participant 3, Theatre).

Many arts providers work in partnership with health and social care providers in the LCR. Although there are clear benefits associated with partnership working, the many challenges facing health and social care providers have hindered the return to in-person provision of arts interventions in health and social care settings. As these institutions have their own complexities and COVID measures to navigate and adhere to, arts provision has not commenced in all health and social care contexts, despite the easing of restrictions:

*A lot of the delays were from partner organisations whose populations weren’t quite ready to go back to in-person, or they didn’t feel ready… because of risk or logistics or a combination of both*… *where the [shared reading] group is usually held [in an NHS Hospital setting]… The consultant who oversees the commission is very, very keen to be careful with the risk, particularly with winter coming* (Participant 8, Shared reading organisation).

Due to strict COVID-19 measures remaining in place in NHS settings, alternative venues may be sought by arts providers to enable face-to-face arts activities to resume:

*At the moment, it’s not possible for us to deliver at the Life Rooms [community NHS service] but we hope that that will change by the time October comes around. If not, we will be speaking to [name of NHS Trust] about whether an alternative location can be found, which is not bound by the same NHS COVID restrictions which would enable us to deliver the sessions safely within the COVID measures that we use at [name of organisation]* (Participant 4, Concert Hall).

Although creative practitioners were not allowed to deliver arts interventions in-person in some health and social care settings following the easing of restrictions, health and social care staff began facilitating group sessions, enabling practitioners to deliver activities via Zoom for small groups of patients or residents:

*In terms of our inpatient sessions with [name of NHS Trust]… Sessions had to be delivered without videos being turned on because of patient confidentiality. That meant that the patients engaged in the session by passing information to the [NHS] staff who used the chat function on Zoom to respond to questions the musicians were asking or where they were asking for feedback or choices about what kind of music to play. So that led to a slightly less interactive experience and a very different way of working for the musicians. But [NHS] staff told us afterwards how much the patients enjoyed and benefited from those activities… In the surveys that some of the staff completed [they] described the sessions enabling service users to interact with others, to express their thoughts, feelings, or ideas, and to experience improved mood… The staff also reported changes in the ward environment describing a happy warm atmosphere with patients feeling calmer, more positive, having more fun… and asking for further sessions. The staff also described how the sessions were a distraction from the chaotic nature of the ward on the day* (Participant 4, Concert Hall).

*[With support from government funding - Department for Digital, Culture, Media & Sport], we were able to do something in some of the villages. We couldn’t go in, but our artists were digitally delivering sessions in the villages bringing together small groups of residents who up until that point had been very isolated within the village environment and not able to socialise on site or have visitors. So, this was one of the early activities that brought them together… The programmes were for six to eight weeks where they might do a programme of ceramics, one group wrote a radio play and recorded it… Some people were just really delighted to be able to get back together again, but with a purpose… [They] perhaps wouldn’t have done it if it hadn’t felt like that as a focus to coming together, and that it was what they needed to perhaps take that step back into socialising again within the villages after such a long time… So, we’re not back to our normal provision really in that programme, and I don’t think we will be for a little while* (Participant 5, Contemporary arts centre).

#### Future uncertainty

Navigating the realities of hybrid work was highlighted as ‘*challenging*’, particularly in relation to uncertainties surrounding winter months and amid concerns of staff burnout:

*There’s that uncertainty of where things might change without your control again, and how quickly then you need to suddenly readapt to, ‘ok everything needs to go back online again’. Even though we’ve got a lot of things in place now [about 25 different documents - we’ve tried to put ourselves in the best place possible to manage any changes and shifts that might happen], it’s still a lot of management and shifting and then the communication, the marketing and comms around all of that having to change… There’s levels of layers of stress and pressure and delivery that have been put on a lot of sectors. I guess the only concern is sustainability of the cultural sector, with that same added pressure of delivery. So as things reopen, we’re expected to be this blended hybrid sector of working online and physical, being everything to everybody. How sustainable is it with not just the funding available, but also the staff that you have? What you don’t want is to burnout essentially… and then people are off unwell, which then has a knock-on effect on the staff that are left there… It’s about how we recover in a sustainable way and at a pace that people can manage* (Participant 14, Photography gallery).

Many arts organisations are facing uncertain financial futures. Much resource is squandered as creative practitioners and small organisations spend time applying for small amounts of funding when they could be using their skills to benefit local communities. Despite showing their value during the pandemic, arts organisations were concerned about future funding, highlighting that funding regimes nationally need to change:

*I think that as a sector, we’re sometimes underfunded and undervalued… With other priorities, like the recovery of the economy, I do worry about the sector being hit by that in a negative way* (Participant 11, Cultural and creative hub).

*I think a world without arts and culture would be a very dark and cold place. What COVID gave us was a real opportunity to shine a light on the value of how important we are. Moving forward, we’ve got to continue to find ways to get that message across in terms of funding and support for engagement, because we are totally reliant on the public purse to survive and to grow as a sector. As with any other industry, we need investment. The hope would be that as we come out of COVID, some of those values of what we’ve seen in terms of impact on society will take us forward and will create more opportunity… We haven’t had the investment in the past for lean days. We’re very project led, it feels like a world of feast or famine, so I think we’ve got to look for more sustainable models of development*…*We have to keep growing and innovating as well, and it’s hard to do that when there is uncertainty around engagement and perhaps even funding. So it feels like we can move forward positively but a concern will be that in time, once that sense of crisis is over in the immediate short term as it has been, that we don’t keep trying to find ways to recognise the value of arts and cultural intervention in people’s lives and to keep investing in that and finding new ways to help the sector grow so it can be more sustainable, and it isn’t grant dependent* (Participant 2, Museum).

*I think the arts and culture sector have played a huge role in supporting people’s health and wellbeing during the pandemic. It seems that that has now been recognised more widely by the public and by the government. I hope that that recognition continues and is met with increased funding for the arts and cultural sector to use its skills and resources to support the health and wellbeing of people in communities. There’s a huge challenge on our hands as the country recovers from the pandemic and is hit with difficulties in terms of NHS spend and social care too* (Participant 4, Concert Hall).

*We know it’s going to take time to rebuild audiences. I think for those of us in the sector who had managed to get a fairly sustainable mixed funding level working for them… it’s worse for us now. But in time, we’ll come back, and we’ll get that balance back. I’m confident of that… It’s how much support I guess the government and the Arts Council and DCMS are going to be prepared to give to us* (Participant 5, Contemporary arts centre).

### Theme 3: Reimagining arts in mental healthcare

#### Culture and arts in social prescribing

As a consequence of the global pandemic and associated restrictions, arts providers and practitioners recognised that ‘*people’s mental health has been damaged*’ (Participant 13, Media art gallery). The pandemic has, however, underlined the importance of community assets, such as arts and culture:

*What I hope has happened is that there’s a greater understanding of how important the arts are to our social wellbeing, to our everyday lives. The understanding of that hopefully translates into a value that can support the arts to grow and to continue to innovate* (Participant 2, Museum).

*I think that arts and culture is going to be needed more than ever… Arts and culture is crucial for people’s mental health. I’m a big advocate of that and I think COVID has made everybody realise this more than ever* (Participant 9, Musician).

*There’s something to be considered around the degree of trauma that everyone’s been through, and the increased numbers and intensity of mental health difficulties… Potentially as well linked to the trauma there might be embodiment of grief in people, both very real bereavement and having lost almost two years of our lives… If you don’t create space for people to find a range of symbolic languages, whether that’s music, dance, theatre, literature, any number of wonderful ways that we connect with how we feel, the thoughts we find difficult to have, and therefore the roots of empathy for one another, I think you’re actually baking in fractures into society* (Participant 8, Shared reading organisation).

According to arts providers and practitioners, solutions beyond medical interventions are essential in order to ensure that people lead healthy lives. As community assets help people feel connected and improve wellbeing, non-clinical approaches are gaining traction for their preventive qualities. As the region emerges from the restrictions imposed, arts providers and practitioners have recognised this transitionary phase as a new era for social prescribing:

*The hope would be that COVID has given us some really good evidence and really good understanding of how arts and culture are absolutely crucial as the social asset to sit alongside clinical intervention/NHS provision. To live well lives, we have to have positive experiences, and the NHS is there to help us when we’re unwell but to remain well in the rest of our lives that’s where arts and culture can play a massive role*… *[One way of achieving] sustainable models of development is to look at our alignment with other health and wellbeing initiatives, and social prescribing is an important one that is evolving, and arts and culture needs to be at the table informing that… What are the future opportunities for [social prescribing]? At a regional level that absolutely will come because I think what we’ve recognised in COVID is that it’s a sum of the parts model in terms of health and wellbeing. Living really fulfilled lives is about having access to things that make you happy when you’re well, not just clinical interventions when you’re unwell* (Participant 2, Museum).

*There seems to be a renewed acceleration of social prescribing opportunities brought about by funding for link workers from the NHS*… *I’m encouraged by sector support organisations such as the Arts Council and the National Academy for Social Prescribing who are now looking at how arts and mental health can be embedded in the NHS long term planning. It will be fascinating to see how that progresses strategically in the future* (Participant 4, Concert Hall).

*As social prescribing is becoming much more spoken about, even though it’s been in the system for many years, 40/50 years now, so many people are exploring more practical ways of how we can use it* (Participant 9, Musician).

Although non-clinical approaches are gaining added resonance within mental healthcare, arts providers and practitioners have raised concerns. As the importance of adequate funding and appropriate training were highlighted, the need to invest in this area is crucial:

*We could really benefit from increased funding from the government around social prescribing and around arts embedded in different NHS settings. I fear that arts is lower on the list of funding priorities, despite its proven benefits* (Participant 4, Concert Hall).

*I was always really concerned about the ethics and also the safety for the art worker and the support that they needed because they could come into quite traumatic situations that they weren’t equipped or trained to deal with. The arts is really good at doing stuff with people and community involvement and it no doubt does help people, but to actually be given this provision as an alternative to health or social care provision is really worrying.* [The people can be vulnerable. You could be with people who are very vulnerable] *It concerns me, not just for the people at the other end but for the people who are delivering it and their security, safety and health as well. I think you should have a situation where you do have professionals who have been trained in either mental health or other health services… who understand the pitfalls and the problems, but you have somebody who has a creative art side working with them… If it was done properly it would be more expensive because you’d have people trained, you’d have professionals working with the artists so you would have two sets of people setting it up (Participant 6, Photographer).*

#### Arts in health settings

According to arts providers and practitioners, NHS systems are under increasing, unsustainable pressure. In the context of rising demand and limited resources, joined up working between the arts and health sectors is crucial to meet demand:

*In terms of arts and wellbeing work, especially mental health, it’s going to be just so significantly important, how we can work better with the health sector to ensure the huge beneficial effects that arts have on wellbeing. There’s a lot of potential actually for developing and really prioritising that work, especially in schools and in areas of high social deprivation, but actually acknowledging that we have all felt something during this time, and that there’s really a lot of work to do to support people… There is going to be a huge increased need for wellbeing services, which are already overstretched, oversubscribed nationally, not only in this city. So, I think there’s an exciting opportunity to think outside the box and to think more about how the arts and health sector work more closely together* (Participant 3, Theatre).

*The concerns are… to make sure that our government is going to come to grips with the need of investment in arts and culture, and how the NHS is obviously in such a terrible state. The idea of having arts and culture as part of the NHS is really, really exciting idea but there are many concerns about it happening in real life, because of obvious reasons with the overload of the NHS, the overload of our GP surgeries, the huge waiting lists. I just hope that the government is going to finally hear that and understand how this is important* (Participant 9, Musician).

Although partnership working was highlighted as crucial to meet rising demand, communication between sectors could be improved in order ‘*to create systems that can be beneficial to people’s mental health*’ (Participant 9, Musician):

*If you start to embed the capacity for people working in direct health care to join up with the voluntary and charitable sector for better outcomes, and if it’s properly resourced, and there’s opportunities built in to communicate well, to have shared platforms, to reflect and learn and change, it’s radical. It’s really, really exciting and it could yield some of that stuff where people can genuinely let healthcare services know what the best form and quality of care is for them* (Participant 8, Shared reading organisation).

*It is absolutely crucial for us to work as a team. There are so many gaps in this networking between people who manage Life Rooms [community NHS service], the facilitators, the practitioners, the participants. We could definitely develop a culture of working much more together… The importance of partnerships, communication and teamwork… is a concern because there’s not enough of it, and so much more needs to be developed on that front. But I am hopeful and thanks to COVID in a way. There’s always innovation coming out of a crisis, and I’m hoping that this is what’s going to push us all into a direction of innovation to make things happen… push ourselves a little further to create systems that can be beneficial to people’s mental health* (Participant 9, Musician).

Although it is recognised that vulnerable populations have been adversely impacted by the pandemic, the important role of arts and culture in supporting public mental health has been recognised by one local NHS Trust:

*One of the Life Rooms members of staff was saying that they were looking at wellbeing top ups, essentially that they were going to talk to big businesses in the city and say because now we can do this on Zoom and we know that this works on Zoom, would you be up for making sure that your staff are enabled to take an hour a day or half an hour a day to do wellbeing top-ups on Zoom as part of their work day. That seems like quite an exciting idea, and yes people in socioeconomically disadvantaged backgrounds really, really, really, really, really have been stuck by this pandemic in a huge way as have really vulnerable and isolated people in terms of mental health, homelessness, migration excetera but actually, there’s a lot of people who have jobs, who maybe wouldn’t consider themselves with those protective characteristics whose mental health has been affected in this way as well. So being able to access something that’s a bit of a top up that gives you a wellbeing buzz that you could do online is potentially really exciting* (Participant 3, Theatre).

For sustainable change to be implemented, however, it is recognised that arts in health is a policy-level issue:

*It can feel very much like you and one care or health partner just make things happen and that’s great. But is there something at a much higher level that can then be embedded and go down the way that just highlights the importance of that work long term and better understanding the cultural role in the context of health* (Participant 14, Photography gallery).

## Discussion

This study set out to examine the impact of the COVID-19 restrictions easing on arts and cultural provision in the Liverpool City Region (LCR), and on the mental health and wellbeing of those whom arts and cultural organisations serve, including those who would usually access arts through formal healthcare routes. Our key findings fall into three main categories: ‘new normal’ arts and cultural provision, new challenges, and the role of arts in health.

Although all restrictions were lifted in England in July 2021, there has not been a ‘clear-cut’ return to conventional provision. Instead, the easing of restrictions denoted the beginning of a transitionary phase, with different arts practices having different priorities following the conclusion of lockdown. For the sake of beneficiary experience, exclusive in-person delivery was essential for activities such as music, whereas hybridity was a priority for other art forms. As the focus for many arts organisations was not on returning to conventional provision, many arts organisations reimagined themselves in order to better serve society’s needs, with some offering provision in alternative spaces, such as outdoors or in libraries, to enhance accessibility and inclusivity.

Following the conclusion of lockdown, beneficiaries and practitioners experienced positive emotions upon re-engaging with others in-person. Renewed access to in-person provision enabled individuals to share emotional experiences, which enhanced feelings of togetherness and a sense of collective healing. There is a renewed appreciation of the humanising and connective power of arts and culture, especially their value for processing the trauma and the emotional impact of the COVID-19 pandemic. The findings further demonstrate the importance of arts activities as ‘steppingstones’. For example, arts providers perceived online provision as an enabler to people engaging in in-person provision, and in-person arts provision as an enabler to other activities. Consistent with previous research (e.g., 19), returning to in-person provision has, however, created many practical and logistical issues. Co-ordinating in-person projects with vulnerable participants presents new challenges, such as updating risk assessments and safety procedures.

In line with previous research, the return to in-person provision was cautious [19], with beneficiaries, arts providers, and practitioners finding themselves readjusting during this transitional stage. As there was a sense of caution and anxiety around re-engaging in-person, arts and cultural organisations and their beneficiaries were mutually concerned to preserve the benefits of digital provision through a hybrid model of delivery. With arts organisations navigating the realities of hybrid work, the possibility of staff burnout was a key concern [19], alongside uncertainties surrounding funding to sustain hybrid provision.

A number of implications arise from the study targeting both the arts and health sectors. First, our findings highlight the importance of regularly seeking beneficiaries’ engagement preferences, especially as there are mixed attitudes towards in-person provision. One arts organisation devised a survey to anonymously seek beneficiaries’ views on engagement methods, planning future provision in light of survey responses. Clear communication on safety measures is also important in order to mitigate some of the anxieties that beneficiaries feel when they balance risks in relation to returning to in-person provision [19]. As the risks remain high, however, maintaining hybrid provision of arts and cultural activity is proving important. Hybrid offerings are seen as critical for the mental health of the region’s population, enabling accessibility for vulnerable groups alongside in-person events that enhance community connectedness. As digital provision has demonstrated its potential to improve access and inclusion for vulnerable groups, investment in accessible arts and culture will be essential as the country emerges from the pandemic, with funding for digital development prioritised. As the health sector continues to grapple with the ongoing impact of the COVID-19 pandemic, it is timely to consider the full gamut of options available to help people recover and reconnect [3]. With the effects of the health crisis likely to be long-lasting, greater investment in cross-sector collaborations will be necessary to help alleviate pressure on the NHS. Cooperative partnerships between the arts and health sectors have been shown to be vital and incorporating digital provision into partnerships between arts and health has strong potential for future sustainability. As non-clinical interventions are gaining traction for their preventive qualities, NHS funds could be redirected towards such provision. For example, salaried NHS posts for artists and creative practitioners would be an important step forward alongside appropriate training for practitioners. Although the roll-out of social prescribing in England prior to the global pandemic reflected an attempt to strengthen lines of referral between the health and arts sector [8], this public health scheme will be more critical than ever in the aftermath of the pandemic.

The reported research has limitations. First, as the sample comprised creative practitioners and service providers of arts and culture from the LCR, the findings reflect the experiences in this geographic area. Second, our sample includes providers and practitioners from civic institutions to grassroots enterprises. Although this broad range provides a breadth of different experiences and perspectives, it may limit the specificity of our findings. Interviews with arts providers and practitioners were conducted between August and October 2021, shortly after the conclusion of lockdown in England. It is possible that these interviews are time sensitive, as many people were at the height of their vaccine immunity at this stage, and practitioners and beneficiaries may have been more willing to return to in-person provision at this point in time. Future research should be conducted with vulnerable groups to explore engagement patterns following the changing of the seasons, especially as restrictions were reintroduced in England during December 2021. Finally, our understanding of the impact of renewed access on beneficiaries has been outlined by arts providers and practitioners, rather than beneficiaries themselves. While the arts providers and practitioners are well placed to reflect on the impact of such provision on customary beneficiaries, who regularly participate in their programmes, capturing the views of beneficiaries themselves will be key to fully understanding the significance of the renewed access.

Through the perspectives of arts providers and practitioners, this paper offers a nuanced understanding of the diverse experiences of the transition to renewed access and/or hybrid provision. As part of a move towards place-based care, the role of arts and culture in providing stigma-free environments to re-connect the vulnerable and isolated is more critical than ever. As the value of creative health partnerships, which harness the collective power of arts in collaboration with health and social care services, was illustrated, mobilising the transformative power of arts and culture to meet the mental health and wellbeing needs of individuals and communities through co-operative cross-sector partnerships and public health schemes, such as social prescribing, is vital. By demonstrating the positive impact of renewed access, this study contributes crucial evidence in support of building back a vibrant arts culture in LCR better oriented towards mental health.

## Electronic supplementary material

Below is the link to the electronic supplementary material.

Supplementary Material 1

## Data Availability

The data generated and analysed during this study are not publicly available as the data collected are sensitive. The data are available from the corresponding author on reasonable request.

## Footnotes

1 https://www.gov.uk/government/groups/culture-recovery-board.

2 The app notified people to self-isolate for 10 days (or less depending on how long ago they were in close proximity to the infected person).
