# Supplementary material for: Lifting lockdown: Renewed access to arts and cultural activities
Source: BMC Public Health. 2022 Oct 10;22:1887. doi: 10.1186/s12889-022-14282-7 (PMC9549849; doi:10.1186/s12889-022-14282-7)
Supplement: Supplementary file 1 — Supplementary Material 1 [file 12889_2022_14282_MOESM1_ESM.docx]

**Interview Schedule (Wave 2)**

**Current provision**

To what extent have activities in your organization returned to pre-lockdown provision?

Can you tell me a little bit more about current (in-person) provision?

- How does current provision compare to pre-lockdown provision? Are there any differences? [Prompt: Are participants socially distancing whilst engaging in arts activities? If so, what impact (if any) does this have?]
- How does current (in-person) provision compare to online provision via Zoom (a) during COVID-19 lockdown (b) at the current time? [Prompt: What works better online? What works better in-person?]

To what degree have pre-lockdown audiences/service users/beneficiaries/members renewed their participation in your organizations’ activities (in-person)?

- How do participants feel about re-integrating in-person/engaging with arts activities in-person?
- [If applicable] Why have some participants not renewed participation (in-person)?
- How do you feel about in-person provision at the moment?

How have you communicated renewed activities?

- Have you used different communication strategies for different audiences?

Can you tell me about the hybrid model? How is this working? [if applicable]

- Are some participants opting to continue participating online rather than return to in-person provision?
- Why have you opted for hybrid delivery? / What are the advantages of implementing a hybrid model?

Can you reflect on any successes?

Can you reflect on any challenges?

What has been the impact of renewed access to the arts on participants’ mental health and wellbeing? / What impact does being back together in a shared physical space have on people’s mental health and wellbeing?

Have you undertaken any evaluation activities of either online or renewed in-person provision? If so, can you tell me a little bit about this? [Prompt: Are the same outcomes achieved through in-person and online provision?]

Can you tell me about your partnership with [insert name of health or social care provider]? Has this partnership sustained/stayed the same or changed over the past year? What does cross-sector collaboration and provision look like now?

Have you formed any new partnerships over the past year?

**Role of arts and culture beyond the ending of restrictions in July 2021**

Do you think arts and culture will play a role in supporting people’s mental health and wellbeing beyond the ending of restrictions in July 2021? If so, what will you do to support people’s mental health and wellbeing during this period?

How effectively do you think your organization is planning for future pandemic-like scenarios?

Do you have any concerns for your organization or sector moving forward?

Do you have any additional comments? Is there anything else you would like to say?
